# Magnetic resonance imaging and devices: a mesmerising combination

**DOI:** 10.1007/s12471-014-0562-8

**Published:** 2014-05-08

**Authors:** C. P. Allaart, C. C. de Cock

**Affiliations:** De Boelelaan 1117, 1081 HV Amsterdam, the Netherlands

Magnetic resonance imaging (MRI) is the fastest growing standard of care in diagnostic imaging. Because of its unique properties such as noninvasive accurate assessment of left ventricular (LV) volumes and regional LV wall motion abnormalities, quantification of flow and tissue characterisation, MRI utilisation in the US is still growing at approximately 3 % per year [1]. Moreover, indications for MRI may expand due to innovative techniques such as magnetic resonance angiography (MRA) or the use of targeted contrast agents.

This increased usage of MRI is paralleled by an annual growth of de novo pacemakers (>700,000) and implantable cardio-defibrillators (>200,000) [2]. Since many recipients of pacemakers and ICDs have substantial comorbidities, it is estimated that up to 75 % will develop an indication for MRI examination during further life [3, 4].

The hazards of MRI examination in patients with pacemakers and ICDs are a consequence of the techniques used [5]. First the static magnetic field may yield mechanical forces on ferromagnetic components and may have unpredictable magnetic sensor activation. Second, the modulated radiofrequency (RF) field may cause heating of cardiac tissue adjacent to the lead tip, may induce life-threatening arrhythmias and may interact with the device leading to over- or under-sensing. Third, the gradient magnetic field may induce life-threatening arrhythmias or may cause over- or under-sensing. Finally, combined field effects can alter device function or lead to electronic reset. Electrical reset may have major implications since it can cause pacemaker inhibition or induce fatal tachyarrhythmias. Currently, MRI conditional CIEDs have become available in which the risk of an electronic reset is significantly reduced by adaptations in the device. Moreover, lead design has been improved to avoid tip heating.

In this issue of the *Netherlands Heart Journal* Van der Graaf and co-workers give an excellent overview of the current status of MRI in patients with cardiac implantable electronic devices (CIED) [6]. They underline that although safety in these patients who are scheduled for MRI examination has traditionally been regarded a major issue, the recently published European Society of Cardiology (ESC) guidelines express a different view [7]. These 2013 guidelines state that MRI can be safely performed irrespective of the properties of the CIED, the type of MRI examination or patient characteristics such as presence or absence of underlying rhythm, as long as safety restrictions are met. Differences in risk between chest and non-chest MRI are not mentioned. Moreover, the absolute number of patients on which these guidelines are based is limited. A total of seven studies each report on small numbers of chest MRIs. For example, in the study by Sommer and co-workers on MRI in 51 patients only 5 had chest scanning, and Naehle and colleagues reported on 18 CIED patients but only 8 patients had examinations of the chest [8, 9]. In a larger study, Mollerus evaluated 127 MRI scans in 103 patients of which 62 were chest MRIs [10]. No threshold elevation was found, but both sensing amplitudes and pacing impedances significantly changed. By far the largest study was conducted by Nazarian and co-workers in 438 patients with pacemakers and ICDs [11]. In this study, pacemaker-dependent patients with an ICD were excluded. In 89 patients chest MRI was performed. Three of the 438 patients had an electrical reset (power-on-reset) leading to occasional pacing inhibition in 2 patients. There were no symptomatic events in these non-pacemaker dependant individuals. In the studies mentioned CRT devices are strongly underrepresented.

Thus, current guidelines concerning MRI in patients with CIED are based on a limited number of relatively small studies in patients with a wide range of devices and leads, typically excluding pacemaker-dependent patients as well as patients with CRT devices. The evidence for safety of chest MRI is based on less than 200 patients and potentially associated arrhythmias are still reported. Although the recent guidelines have become more lenient and major complications have rarely been reported, risk assessment in patients with CIED is not straightforward. We would therefore advocate a prudent approach in scheduling patients with CIED for MRI examination, especially those with older type or CRT devices and when using newer (3T) MRI suites. MRI provides mesmerising image quality, but mesmerisation of CIED should be avoided.
